# Qualitative Research for One Health: From Methodological Principles to Impactful Applications

**DOI:** 10.3389/fvets.2020.00070

**Published:** 2020-02-18

**Authors:** Chris Degeling, Melanie Rock

**Affiliations:** ^1^Faculty of Social Science, Australian Centre for Health Engagement, Evidence and Values, School of Health and Society, University of Wollongong, Wollongong, NSW, Australia; ^2^Department of Community Health Sciences, Cumming School of Medicine, University of Calgary, Calgary, AB, Canada

**Keywords:** interdiscipinary studies, social values, research design, veterinary medicine, social sciences

## Abstract

The One Health concept has inspired a rich vein of applied research and scholarly reflection over the past decade, yet with little influence from qualitative methodologists. With this overview, we describe the underpinning assumptions, purposes, and potential pitfalls of data collection techniques and methods of data analysis in key qualitative research methodologies. Our aim is to enhance One Health collaborations involving qualitative researchers, veterinary epidemiologists, and veterinary economists. There exist several distinct traditions of qualitative research, from which we draw selectively for illustrative purposes. Notwithstanding important distinctions, we emphasize commonalities and the potential for collaborative impact. The most important commonality is a shared focus on contextualizing human behavior and experience–culturally, economically, historically, and socially. We demonstrate that in-depth attention to context can assist veterinary economists and epidemiologists in drawing lessons from the implementation of policies and programs. In other words, qualitative researchers can assist One Health teams in distilling insights from “success stories,” but also from adverse events and unintended consequences. As a result, qualitative researchers can contribute to One Health research and policy discussions by formulating more accurate and contextually-relevant parameters for future quantitative studies. When performed well, qualitative methodologies can help veterinary economists and epidemiologists to develop impactful research questions, to create more accurate and contextually-relevant parameters for quantitative studies, and to develop policy recommendations and interventions that are attuned to the political and socio-cultural context of their implementation. In sketching out the properties and features of influential methodologies, we underscore the value of working with seasoned qualitative researchers to incorporate questions about “what,” “how,” and “why” in mixed-methods research designs.

## Background

Over the past decade, the One Health concept has gained traction in policy, practice, and research. Thus far, economics has proved to be the social science with the most influence in One Health research and related applications (1). Economic evaluations certainly can complement epidemiological models in One Health research. Even so, social sciences besides economics could also play important roles. Here we are thinking about disciplines such as anthropology, geography, philosophy, psychology, and sociology (2). Within these social sciences, qualitative research has deep roots; and within professional fields that focus on human health, such as medicine, nursing, and public health, qualitative research has become increasingly integrated (3, 4). This article, therefore, seeks to elevate the place of qualitative research in One Health scholarship and applications.

The goal of qualitative research is the development of concepts which help us to understand human behavior and social phenomena in their natural settings, giving emphasis to the meanings, experiences, and views of those implicated and effected (3, 5). The conditions and systems that produce health in individuals and their populations have long been a focus of qualitative researchers. Public health problems (including those involving other species) are increasingly derived from interactions between human behavior and social and technical systems. Veterinary public health researchers, epidemiologists and policymakers can benefit from integrating insights from qualitative research into their understanding of animal health, not least to improve policies and interventions for the management of the incidence and risks of disease in animals and the provision of related services (6, 7). Disciplines such as sociology, geography and anthropology have made significant contribution to our capacity to understand public health problems, and they solutions that are both novel and contextually-appropriate.

We have written this article with veterinary economists and epidemiologists in mind, especially those who may wish to collaborate with qualitative researchers. Acknowledging that other disciplines relevant to One Health such as public health and the environmental sciences could benefit from greater engagement with social science methodologies, for the purposes of this special journal issue we wish to assist veterinary economists and epidemiologists with studies that combine quantitative with qualitative methodologies. Our overall aim, therefore, is to impart foundational knowledge regarding qualitative methodologies and applications. The references that we cite, in addition, could provide additional guidance for readers who wish to delve more deeply into qualitative methodologies and their potential for research regarding One Health. To begin, we call attention to the importance of interpretation and meaning in qualitative research. This emphasis leads us to compare two popular approaches, namely **framework analysis** and **grounded theory**. After outlining some of the key features of each, we distinguish methodologies from methods. Then we describe some popular methods for collecting and creating qualitative data; these include interviews, storytelling, and texts. We provide examples in which such data have been employed, synthesized and interpreted to enhance veterinary findings, so that they are better attuned to their political and socio-cultural context.

Considerable expertise and skill are required for impactful research that combines qualitative with quantitative methodologies. Integrating qualitative studies into larger mixed-methods requires robust research design and processes. Experience with and expertise in the method used is a must; insufficient expertise and practical experience can lead to a superficial and even harmful impacts. Because analysis begins at the same time as data collection in most qualitative social science research, in this article we have foregrounded a descriptive summary of different types of data analysis over the standard toolbox of qualitative data collection techniques found in methods textbook. Our purpose is to highlight that robust research processes require that the technique used for analysis should be determined as part of the initial research design—and not chosen as an afterthought once data collection is underway. Of note, researchers should design their studies from the outset with an approach to analysis in mind. By implication, veterinary economists and epidemiologists should include qualitative researchers from the outset if they intend to undertake a qualitative investigation as part of a larger multi-method study. Moreover, even as we invite our colleagues in veterinary economics and epidemiology to consider including qualitative researchers on their teams, we caution against superficiality. In our experience, qualitative researchers are best positioned to contribute when One Health projects have been designed collaboratively, with them and with communities.

## Foundations and shared Assumptions in Qualitative Research Methodologies

While both qualitative and quantitative research can be used to investigate similar topics of relevance to veterinary economics and epidemiology, each will address a different type of question. In relation to the use of an animal vaccine, for example, a quantitative study could determine the proportion and demographic characteristics of owners who give the vaccine to their animals over a set period of time. Similarly, questionnaires that try and capture people's intentions can test the relative importance of different factors thought to be important to their decisions through the statistical analysis of responses to a series of standardized closed questions [See (8, 9) for example]. However, unless participants have the opportunity to respond to open questions in their own words then the research cannot capture novel and unanticipated information. To answer questions about why some owners do not vaccinate their animals, a qualitative study could explore both anticipated and unanticipated factors that might influence people's choices, such as their beliefs and values. Anthropological or qualitative studies can answer questions such as “What are X and Y? How do X and Y vary in different circumstances, and why?” (10, 11).

Research methods are the tools or techniques used to collect or analyse data to answer questions and to achieve objectives. In contrast, research methodologies are accounts of philosophical principles and theoretical underpinnings of the approach and methods being used in a study (12). Qualitative researchers tend to take an interpretive, naturalistic approach to the subject of the inquiry. The methods and methodologies used in qualitative research accept that there are a range of ways that we can understand and explain the world we live in, such that the focus of inquiry is on discovering and understanding the perspectives and experiences of those being researched (13). Developing this understanding moves beyond descriptive reporting. The application of theoretical knowledge, in qualitative studies, enables specific cases to be seen and described in more abstract terms (12). Systematically comparing within and between different cases can yield more detailed insights about particular contexts or practices, as well as generalizable knowledge about specific types of social phenomena that occur in different settings (14). As a general but by no means absolute rule, qualitative research methods and methodologies share some broad orientations which include: (i) a commitment to naturalism; (ii) a focus on understanding; and (iii) a flexible approach (3, 13). All of these assumptions are shared with observational methods in epidemiology (15, 16).

**Naturalism** is important because context influences people's behavior, and the beliefs and values that underpin their behavior. As we elaborate below, ethnographic methods are perhaps most committed to understanding how and why people behave the way they do in “real life” because the researcher spends extended amounts of time in the study setting, observing and partaking in the every-day activities, periodic events, and conversations. Having someone observe what is happening in the social world will inevitably have some impact on what is being studied. But this limitation can be managed through reflexive research practices (17, 18). Being committed to understanding how real life works in a specific place and time or setting creates valuable information because researchers who are familiar with and knowledgeable about how people live and the constraints and choices they face are more likely to grasp how social context has impacts on people's behavior.

As an example, the anthropologist Lyle Fearnley has described how, for duck and poultry farmers near Poyang Lake in Eastern China, diseases that afflict their birds are not considered not so much as a global health threat, but as a by-product of the new agricultural systems and modes of intensive farming that currently underpin the “family business.” Now recognized as a key hotspot for highly pathogenic avian influenza (HPAI) emergence, poultry farmers have set up enterprises around local wetlands raising, slaughtering, and selling flocks of “farmed wild birds” such as swan geese. These “farmed” wildlife go out onto the lake each day to graze and hence they mingle with flocks of migratory birds, which number in the millions, potentially providing a bridge for the transfer of viruses between wild waterfowl and domestic poultry (19). Moreover, local duck farmers are unable to sell or transport their birds during HPAI outbreaks, leading them try to reduce feed costs and maintain the capital invested in their flock by free-grazing them on the lake. Paradoxically, these practices increase the local risk of viral transfer and spill-over (20).

**Maintaining a focus on understanding** the world (or the part of it we are interested in) from the perspective of the participants creates a critical stance from which social scientist can remain skeptical of widely-held assumptions and received wisdom. Qualitative research methods move beyond simply attempting to describe social phenomena to characterizing how different objects, processes or goals acquire meaning and significance. The views that are most important are those of community members, and not those of the researchers. Thus, the best line of questioning might not be: “Why don't farmers implement biosecurity protocols?” but “How do farmers make decisions about maintaining biosecurity, or not?”; and “What kind of evidence is used to make these decisions?” These open and non-judgemental lines of questioning start from the assumption that people have good reasons for their beliefs and actions, and part of the job of the researcher is to understand their priorities, their goals, and the constraints that shape their choices and behaviors. Qualitative researchers are not simply a reporter, taking notes and writing down stories. As qualitative researchers, we must also analyse those accounts, and link the empirical findings with a theoretical understanding of what happens during fieldwork.

For instance, in a detailed body of work the geographer Gareth Enticott has described how farmers in the United Kingdom rationalize expert advice and biosecurity protocols for managing bovine tuberculosis (bTB). These farmers tend to view bTB in their herds as locally contingent, down to luck and matters of spatial ordering. Twenty years ago, the Department for Environment, Food, and Rural Affairs (DEFRA) guidance for bTB prescribed spatial separation between herds on adjacent farms, limiting introductions of new stock, and keeping badgers (as a suspected reservoir host of bTB) out of barns and yards. Drawing on scientific studies, DEFRA also proposed that reducing the size of badger populations (through culling) in pastures was unrealistic—and likely to be counterproductive by causing perturbations in badger territories and populations. Evidence supporting this assessment was generally rejected by farmers as being simplistic and impractical, because it did not account for the practical experiences of farmers (21).

Instead, as Enticott describes, farmers generally maintained two ways of understanding the incidence of bTB in their herds: (1) as being predictable; and (2) as being unpredictable. Whenever bTB emerges, this paradox allows farmers to avoid blaming themselves, to claim a sense of stewardship over the countryside, and to sustain a view of themselves as still being in control (22). For farmers attempting to manage bTB in their herds, agricultural space should not be left open to negotiation. Space could not be ordered in the way the DEFRA guidelines proposed, to prevent badgers moving in and out of their farms, so the preferred solution was to limit the number of badgers inhabiting local landscapes through culling (21, 23). At the same time, ongoing controversy about the validity of the scientific evidence meant that policymakers in Wales and the UK formed partnerships with different stakeholder groups and policy actors, which resulted in different legislative responses in these jurisdictions (24).

**Flexible research strategies**, as demonstrated by Enticott's (21–24) work above, are often needed to complete rigorous qualitative studies. Careful planning remains important, but the researchers need to adapt their plans as data is produced and analyzed, and as policies change. Qualitative studies may involve several interleaving and iterative research phases. Qualitative researchers might start with a literature or scoping review of textual sources, which then progress out into the field to undertake periods of participant observation, individual interviews, and information exchange and engagement with communities and policy stakeholders.

## Methodological Approaches to Analysis in Qualitative Health Research

As noted in the introduction, in this article we have foregrounded a descriptive summary of different types of data analysis over the standard toolbox of qualitative data collection techniques found in methods textbooks. We have taken this step to highlight that in most qualitative studies, analysis begins during data collection, so as to iteratively shape ongoing processes of inquiry. This approach permits the refinement of research questions, the development and testing of theoretical propositions, and the pursuit of new insights or lines of inquiry as a study evolves. Importantly, combining data collection and analysis also allows researchers to look for divergence; that is, examples of talk or events that run counter to initial expectations and the emerging findings. Insufficient attention to identifying and exploring the full range of alternative explanations is a common flaw in qualitative studies undertaken by poorly-trained or inexperienced investigators. Close examination of deviant or negative cases is an essential step that allows further refinement of the research questions, study design, and interpretation of the findings. Maintaining respectful skepticism or some form of continuous, critical and reflexive analysis of what the researcher is seeing and being told is an essential component of rigourous practice in qualitative research.

Interview transcripts, other texts, images and notes are the raw data—explanations and insights are developed through iterative rounds of interpretation (25). No matter the method of data collection, qualitative researchers often maintain have a separate note book or electronic document to jot down ideas, impressions, and early interpretations (26, 27). As well as opening up new lines of inquiry, as an understanding of the social phenomenon being studied begins to emerge, this practice enables qualitative researchers to remain aware of their own positions. Self-awareness is the foundation for continuous critical reflection about social positions and potential biases related to various aspects of the research process (26). Where members of a research team have in-depth knowledge and expertise in the field being studied, the process of analysis and interpretation can also draw on their own understanding of daily life and important events, along the lines of an informal ethnographic study (28, 29).

In qualitative studies, researchers set out to identify commonalities and differences within the data set, before focusing on relationships between different parts of the data. The overarching goal is to draw descriptive conclusions, explanatory conclusions, or both, clustered around emergent or pre-determined themes. Most qualitative research involves some variant of content analysis. Veterinary economists and epidemiologists will benefit from realizing that there exist some key differences between commonly used approaches in qualitative research. Each is underpinned by different sets of assumptions about how we experience the world and communicate meaning which shape the process of analysis (12). In what follows, we describe different stages of analysis commonly found in qualitative research projects. Then, for illustrative purposes, we attend to key differences between common inductive and deductive approaches, with reference to **Grounded Theory** and **Framework Analysis**.

### Stage 1: Transcription and Familiarization

Data collection in qualitative research typically results in an indexed collection of textual materials (reflective notes, policy documents, news media, etc.) and/or audio-recordings of what people say in interviews or when interacting with each other in a group. Audio-recorded data is usually transcribed into textual form to aid in analysis. The first stage is for qualitative researchers to familiarize themselves with each interview using the audio recordings, transcripts and any contextual or reflective notes that were recorded at the time. This process of familiarization requires reading and re-reading the transcripts, and potentially listening the audio-recordings. Through this labor-intensive process, qualitative researchers begin to grasp which themes and concepts are most likely to be important for the purposes of interpretation, and how these preliminary insights might be relevant to the phenomena and context being studied (30, 31).

### Stage 2: Coding

After they have familiarized themselves with the data, qualitative researchers typically review transcripts and generate a set of codes (a label that describes a concept or analytic category). This way of coding comprises a first step in systematically interpreting a qualitative dataset (32). The aim of coding is to classify all of the data so that segments can be compared systematically with other parts of the dataset. Qualitative researchers often work alongside one another at this stage. When two researchers independently code a handful of transcripts—annotating them with notes—that helps promote internal reliability (33). Qualitative researchers' codes may be generated **inductively**—that is, derived gradually from the data—or predetermined and applied **deductively** as a way of approaching the data, either at the beginning or part way through the analysis. Coding is thus an interactive and iterative process—sometimes described as being in dialogue with the data.

**Framework Analysis** has gained popularity as a **deductive approach** to qualitative health research. When undertaking a deductive analysis, the codes are usually pre-defined by an existing body of knowledge; and in framework analysis, the codebook stems from policy-relevant questions. This analytic strategy emerged in the United Kingdom to advance policy-focused research, wherein the study objectives are largely determined in advance, based on the requirements of knowledge users (34). A set of pre-set questions facilitates a process of deduction through constant comparison, by organizing qualitative data that correspond to these questions within an organizing matrix (35). Codes can still emerge from the data to capture unexpected themes—such that this approach to analysis allows qualitative researchers to accommodate both predetermined and unanticipated objectives for the research, so long as the unanticipated objectives have relevance for the policy issue at hand. Framework Analysis was explicitly designed so that procedures and outcomes can be assessed by people other than the qualitative researchers who designed and conducted a given study. This commitment to producing data that can be *a priori* organized and readily analyzed by other researchers or policy actors limits the use of the Framework Analysis to relatively homogenous data-sets. In a study guided by Framework Analysis, all of the data must address similar topics or key issues; otherwise, researchers would not be able to apply a codebook to the dataset. For these reasons, data collection for studies that employ Framework Analysis tend to be more structured and directive than in qualitative research that is inductively oriented, aimed at advancing social theory, or both (36). Examples of Framework Analysis in veterinary research include a study of disease management decision-making in aquaculture in Bangladesh (37); the policy imperatives driving poultry vaccination in Indonesia, Thailand and Vietnam (38); and the attitudes and practices for anthrax prevention amongst farmers in Zimbabwe (39).

For more **inductive studies**, such as those based on **Grounded Theory**, qualitative researchers go backwards and forwards between recruitment of participants, fieldnotes, interview transcripts, and the processes of conceptualization, thereby trying to make sense of the data as it is generated or collected (40, 41). In the process, qualitative researchers identify a set of cases for purposes of comparison. Based on constant comparison across cases, they develop explanatory propositions, and then they examine additional cases to confirm, discard, and refine these propositions.

Grounded Theory emerged from the ethnographic tradition in American sociology, including the sociology of health, illness, and medical knowledge. Hence studies based on Grounded Theory aim mainly at generating insights about social life in the first instance, as compared with providing practical advice to guide policy and programming, as in Framework Analysis. Impactful insights about policy and programming, however, can certainly emerge from studies based on Grounded Theory. Examples of Grounded Theory in veterinary research include studies on how pastoralists make decisions about sick chronically sick animals in Cameroon (42); the effect of compensation on farmer attitudes to exotic disease reporting (43); the politics of veterinarians and feed-store vendor control of access to antibiotics in dairy farms in rural Peru (44); and accounting for variation in people's responses to the death of their animal companions (45). In addition, veterinary economists and veterinarians might wish to compare a Framework Analysis of aquaculture in Bangladesh (37) with an inductively-based analysis, also of aquaculture in Bangladesh (46), that resembles Clarke's take on Grounded Theory (41).

As illustrated by Grounded Theory, the hallmark of qualitative research based on inductive reasoning is the gradual development of a novel set of codes based directly on a specific project. The analytic process usually proceeds through constant comparison, in which each item is checked or compared with reference to the dataset as a whole to establish analytical categories (47). Initial coding should be open—such that an interpretive label or category is applied to anything in the data that might be relevant. Codes can refer to anything, for example events or institutions, values, emotions or how a participant behaved or how an interviewer felt during an interview.

Inductive coding must go beyond fine-grained description to be inclusive of variation and to generate novel insights. Qualitative researchers who rely mainly on inductive reasoning, therefore, pay close attention to the unexpected so that the developing analysis is challenged and alternative propositions are considered. Anomalies need to be explained—the key point being that categories may added at any point of the study, even during the writing and revising process, to reflect as many of the nuances in the data as possible (41, 48). When qualitative researchers become immersed in the research setting or in communities, for example, they often witness interactions or participate in events that seem surprising. The sense of surprise arises because first-hand participation in the social life of a community has revealed phenomena that depart from received wisdom, or that highlight the researchers' unconscious biases. Rather than suppress or ignore such surprises, qualitative researchers who privilege induction in their studies tend to treat surprising interactions or events as keys to understanding. Many will articulate (“crystallize”) insights of relevance to the entire study out of an initial sense of surprise (49).

The anthropologist Michael Agar, therefore, argued that abduction is the analytic partner of induction in qualitative studies. By abduction, he meant the surprises that qualitative researchers absolutely must take “seriously as a signal of a difference between what you know and what you need to learn to understand and explain what just happened” (29, p. 64). Gradually, through a process of conceptual crystallization, qualitative researchers distill key characteristics of communities or influences on networks. At the same time, qualitative researchers may leverage marked similarities and differences within the dataset to generate more refined typologies, to interrogate important concepts, or explore how categories that manifest in the dataset relate to phenomena in social life (49). Consequently, qualitative researchers who emphasize inductive reasoning often need to collect or generate additional data for purposes of comparison, especially if new or unanticipated ideas come to the fore during the iterative process of fieldwork and analysis. Hence a flexible approach is important not only for qualitative researchers themselves, but also for inter-disciplinary teams that include qualitative researchers. The more experienced the qualitative researcher, the more a qualitative researcher will tend to rely on abductive and inductive reasoning, which gives rise to a greater need for flexibility than in deductively-driven qualitative methodologies, such as Framework Analysis. Nonetheless, qualitative researchers' need for flexibility can be challenging to manage in mixed-methodology research teams (3, 50).

### Stage 3: Abstracting the Main Findings

The outcome from the generation and application of codes to the dataset is a taxonomy that describes and interprets the social phenomenon of research interest. In qualitative research based on deductive reasoning, analysts can usually rely on a detailed codebook derived pragmatically from policy-related debates, which they adapt and update as the project progresses. By contrast, when coding based mainly on inductive reasoning, qualitative researchers typically create all or most of the codes themselves, sometimes *de novo*, and sometimes in relation to previous scholarship of a theoretical nature about social life. Experienced researchers who specialize in qualitative methodologies vary in the extent to which their analyses revolve around applying a detailed codebook to their datasets (49).

As qualitative researchers gain momentum and analytic purchase on the research questions, whether through deduction or by combining induction with abduction, they shift their efforts toward clarification. Visual aids such as diagrams, maps and tables may assist in depicting key codes and insights to include and elaborate in subsequent analyses (33). Informed by the analytical and theoretical ideas developed during the research, qualitative researchers then invest time in refining the emergent insights and codes, typically by regrouping them as subcategories under broader categories (also known as “themes”). Then they may select key themes for further investigation. Depending on the depth and richness of the data, the lessons learned through this process may apply well-beyond the description of specific communities, settings, or networks. Qualitative researchers often refer to “transferability,” as compared with generalizability, to signal their capacity to generate insights with implications for policy and planning. For example, qualitative researchers may be able to illuminate the reasons why something is happening, to the extent of predicting how different groups might respond to a situation, or identifying dysfunctional dynamics or constructive disruption within policy-relevant organizations and economic systems.

### Stage 4: Interpretation of the Results

As is the case when employing quantitative research methods, the results or findings from qualitative studies need to be contextualized within the outcomes of previous qualitative and quantitative research. This can include a summary of the similarities and differences with findings of other research studies, including reflections on the relevant socio-historical and scientific context. For **deductive studies**, such as those that employ **Framework Analysis**, for example, the interpretation of the results might also include a clear set of action-guiding or policy recommendations informed by the attitudes, beliefs and values of participants (the people the issue effects) By comparison, the results of more **inductive studies** should include some discussion of the relationship between the results of the current study and established theories about social life (3). Within these efforts to describe the broader significance of the study outcomes it is also important to describe any limitations to the way the study was conducted or the generalisability of the study findings.

## Data Collection Methods and Sampling Considerations in Qualitative Research

The types of data that can be used in qualitative research include what people say, what they do, and how they interact with each other and the world around them (3, 25). Materials and data can include observations made by the researcher and other media through which people communicate information, such as talking, images, symbols, and textual sources; for example, policy documents, scholarly and gray literatures, signs, and posters and the contents of social and news media. Key techniques for collecting data in qualitative research include: interviews, focus groups, and other group-oriented research and engagement practices such as storyboarding and deliberative methods.

A key difference between quantitative and qualitative methodologies is the way in which the study sample is conceptualized. The aim in quantitative studies is to produce a sample that is in some way statistically representative of the whole population of interest. Consequently, a probability sample is typically used. In qualitative work the sample size depends on the study aims—what you are expecting the data to do in terms of answering the research questions. Accordingly, most qualitative research uses purposive sampling. This entails explicitly selecting participants who can generate the data appropriate to meeting the research aims and objectives; while also being able to be identified as being “representative” to those who will use the research. Sometimes mixed sampling strategies (involving quotas of different types of people likely to have different perspectives) are used to generate information-rich cases where cultural variables are likely to be important analytically.

The most methodologically convincing criteria for ensuring study rigor is to sample theoretically; that is, until data saturation where no new insights are emerging and all themes, categories and variations are fully accounted for. But this level of detail is rarely achievable given the almost limitless amount of resources required to truly achieve theoretical saturation (51). So, the most practical and pragmatic answer to the question: “how many of what types of people should make up the sample for a qualitative research study?” is “however many of the range of different types of people who will be credible to users of the research.”

### Interviews

Interviews provide an account of an individual's experiences, thoughts and perspectives. Interviews can be with individuals, or with small groups where the focus is on individual perspectives and not interactions within the group. It is important that interviewers try to be sensitive to the language and concepts used by the person(s) being interviewed. The aim is to explore in depth the topic being discussed and not, as is often the case in poorly-designed qualitative studies conducted by inexperienced investigators, to undertake “tick-box” exercises on a predetermined list of possible factors. To ensure they are not recording an inaccurate or superficial account, interviewers must actively check they have understood the participants' meanings, rather than relying on their own assumptions. Interviews should be conducted at the convenience of the participants. Given that the setting of an interview inevitably affects the content and what the qualitative researcher will be able to observe and to infer, qualitative interviews tend to take place in setting that is familiar to the participants, such as at their workplace, home, or a nearby public space. Overall, qualitative interviewers seek to make the participant feel as comfortable as possible.

Interview-based studies in qualitative health research do not sample seeking to attain statistical representativeness. That is because statistical representativeness is not necessary when the objective of the study is to understand social processes. Sample sizes are determined by factors such as the depth and duration of the interviews, and whether or not data saturation is being reached and no new themes on insights are emerging (52). Systematic, non-probabilistic sampling is favored because the purpose is to identify specific groups of people who live in circumstances or possess characteristics relevant to the social phenomenon of research interest. This approach to sampling can allow qualitative researchers to include a wide range of types of people who have perspectives relevant to the research, while also recruiting participants with access to relevant sources of knowledge and social networks. Alternatively, as in criterion-intensive sampling, qualitative researchers may recruit participants who represent a narrow range of characteristics and positions.

Qualitative researchers usually design an interview guide or schedule that contains a list of core questions that approach the topic of interest from different angles. Unlike the highly structured questionnaires used in quantitative interviews, in **semi-structured interviews**, most of the questions are open ended so that the participants can respond in their own words and their ideas can be explored in more detail. The type of analysis being conducted should also shape the questions. In **deductively-driven studies**—where at least some of the analytic codes are predetermined as part of the study design—the questions put to participants can be framed much more tightly around the subject or issue of research interest. By contrast, with **inductive studies**—where the analytic codes, themes and findings of interest to the researcher emerge through their interactions with the collected data—the qualitative researchers' questions need to be sufficiently broad to cover a wide range of experiences but narrow enough to allow a focus on exploring the participants perspectives and experiences (3). Independent of whether the study is **deductive** or **inductive** in orientation, semi-structured interviews need to be attuned to the participants' responses and perspectives. Therefore, the order of questions can vary, as can the content and focus of different questions as the researcher attempts to grasp what the person being interviewed means—often using the same terms and concept as the interviewee when adding supplementary questions. Also, qualitative researchers should regard silences, evasive responses, and apparent discomfort as meaningful. For instance, participants may feel reluctant to criticize local power-brokers directly or even obliquely.

**In-depth interviews** have less pre-set structure than semi-structured interviews. In many instances, qualitative researchers conduct in-depth interviews to explore, in detail, just one or perhaps two issues (3). Alternatively, qualitative researchers may conduct in-depth interviews that chart the life-course of participants, whether as influential actors, or as “ordinary” people whose life-stories illuminate social life (53). Ethnographic interviews (54) combine immersion in the study setting and first-hand interactions with participants (see section below). Usually ethnographic interviews take place with one participant at a time, but not always. When conducting ethnographic interviews, qualitative researchers usually go to where the person or group being studied does the activity that is of interest to the study and to talk to them in this context. The idea is to follow people in their every-day setting, while they are performing every-day activities, asking them questions about what they are doing and why (when necessary) along the way (55, 56). Observing people as they take part in activities and questioning them in the settings of daily life can draw attention to important details about the context and their behavior. Overall, an participatory approach to interviewing people can assist qualitative researchers in understanding how local meanings and practices reflect and reproduce key structures in social life (57).

Studies conducted by the authors of this paper, as well as by others, demonstrate some of the type of knowledge that can be gleaned from semi-structured and in-depth interviews. For example, we have published articles on urban dog-walking in Australia and Canada, based on interviews that all began by asking participants about how they took care of their dogs (58, 59). These articles followed on from a qualitative study of how people cared for dogs and cats with diabetes, which also involved ethnographic interviews. These interviews revealed that the people interpreted how their pets were faring by recognizing these animals as sentient selves (60, 61). The participants who lived with diabetes themselves regarded their diabetic pets as being akin to them, even as a kindred spirit in one memorable example. Focusing on a similar line of inquiry, Vanessa Ashall and Pru Hobson-West conducted 21 semi-structured interviews in the UK with people who put forward their pet animals as donors for canine and feline blood banks. Rather than being founded purely on altruism, owners' motivations included a desire to display their identification of their animal as a member of their family, while at the same time assuaging their guilt for not volunteering themselves as donors for human blood banks (62). Studies like these illustrate how knowledge of the motivations, beliefs and understandings of animal owners around specific disease conditions or types of clinical practices can enrich epidemiological work that covers the same area, providing context and potentially explaining human behavior, the drivers of demand, and the choices that lay-people make about animal care in veterinary clinical contexts.

Numerous studies involving qualitative interviews have taken place in remote regions and resource-constrained countries. For example, by conducting semi-structured and in-depth interviews with people living on the edge of the Kibale National Park in Uganda, in addition to administering questionnaires, Paige et al. showed that local residents were highly informed about a broad range of zoonotic diseases and risks pertinent to their local area; and they also highlighted new potential sources and pathways of transmission (63). Drawing on parasitological and epidemiological data on *Taenia solinum* from humans and domestic pigs in a remote region of PDR Lao, an interview-based project led by Kevin Bardosh showed the relationships between alcohol, ancestral sacrifice, and the consumption of uncooked (cyst-infected) pork were central to village life. As a result, health communication campaigns (advocating cooking and better hygiene, for example) had limited impacts on culturally-embedded risk behaviors—highlighting that all interventions need to be adapted to cultural settings (6). These citations are examples of rigorous and impactful contributions from qualitative researchers to the control and prevention of zoonoses (64–67), or the effectiveness of responding organizations (68, 69).

### Participant Observation

By describing daily life, common routines, and unusual events in a community or network, participant-observation can assist qualitative researchers in generating novel insights about social life, and such insights can be germane to veterinary economics and epidemiology. For example, Alex Nading's study of urban households in Ciduad Sandio Nicaragua showed how biosecurity and vector control practices altered social relationships, highlighting that people in particular socio-cultural and geographical contexts interpret disease and disease control measures differently (70). This study would not have been possible without in-depth preparation and first-hand knowledge of the local context. Nading lived within the community at the time, and he spoke Spanish well-enough to conduct interviews in that language and to accompany local people as they worked. For community health workers, who were women and who were charged with intervening directly with residents to improve *Ae aegypti* control, learning about mosquito ecology and the place of Dengue fever in colonial history was the spur for reflection on and political engagement with their own situation. Rather than embrace the goals of the dengue control program, they came to identify with the female mosquitoes. In concert with a lack of scientific rigor and an overdependence on already overworked and strained volunteers, perceptions of lived-connections between community workers and female mosquitos, ultimately contributed to the failure of top-down dengue control programs in this setting (71). Nading's careful ethnographic work shows how a focus on human-animal sociality in specific places can illuminate policies informed by veterinary economics and epidemiology. In this type of social inquiry, the people and environments in and around specific sites of program implementation are highlighted, thereby yielding novel insight into global health interventions (72).

When under taking participant observation the choice of settings determines the sample (who, where, and what is observed). The choice of venues for the study is therefore critical to how well the data generated will address the research objectives, and the generalisability of any findings (73). As a research practice, participant-observation has strong roots in anthropology, sociology, and geography. Reflexively accounting for the effects of presence of the researchers and their guiding assumptions is key in any study that involves participant-observation (74). To be sure, as discussed earlier in this article, qualitative researchers must seek to identify unconscious biases, and then to move beyond such biases and toward a deeper understanding than what was possible at the outset. Participant-observation by qualitative researchers can bring unconscious biases to light, which may seem paradoxical (29). In other words, participant-observation harnesses the qualitative researchers' own subjectivity as a resource, rather than trying to eliminate or minimize subjectivity as an unwelcome source of bias and an obstruction to scientific knowledge.

### Working With Groups

**Focus groups** are a form of group interview that draws on discussions between research participants to generate data, whereby the researcher acts a discussion moderator. The method is particularly useful for exploring people's knowledge and experiences. Focus groups are especially useful in approaching the study of organizational cultures and the operation of dominant cultural norms and values. In a focus group the researcher explicitly uses interactions between participants as part of the method—a schedule of questions acts as prompts for the group discussion. The assumption is that group processes allow people to explore and clarify their own perspective through explaining themselves to the group and listening to other people's perspectives (75).

Focus group studies can consist of anything between 4 and 50 groups, depending on the aims of the project and the resources available (76–78). Most studies are small in scale and part of a larger multi-method study. To capitalize on people's shared experiences, qualitative researchers usually aim for homogeneity within each focus group such that the sample is comprised of groups of similar participants. A significant limitation of focus group methods is that group dynamics can often work to silence minority voices. That said group work can also actively facilitate the discussion of otherwise unmentionable topics or provide opportunities for otherwise disempowered groups in society to raise issues that are important to them.

As an example, a multi-disciplinary team from the *Dynamic Drivers of Disease in Africa Program* conducted a series of studies of the social and cultural determinant of the incidence in Lassa fever in Sierra Leone, Henipah virus in Ghana, RVF in Kenya, and Trypanosomiasis in Zambia and Zimbabwe. A series of focus groups were conducted to complement a household survey, social mapping exercises and in-depth qualitative interviews with individuals. Focus groups allowed gender, occupation and age specific discussions to take place. These discussions revealed otherwise hidden cultural dimensions of disease risk, capturing sources of knowledge not conventionally considered in disease risk models which enriched analyses with local insights and perspectives based on local knowledges (7). Focus groups were also by Bardosh et al. in the previously mentioned rapid study of the transmission dynamics of *T. solinum* in a remote village in PDR Lao (6). These examples of multi-method field-based studies also show that generalized assessments of disease risks are only the first step. Cost effective and targeted interventions follow from understanding who gets sick, when and where, through engaging with the affected communities.

Focus groups methods can also be used to engage with experts. Victoria Ng and Jan Sargent employed focus groups to identify criteria for the prioritization of zoonotic disease in Canada (79). Representatives of different stakeholders such as animal health professionals, human health professionals and lay-members of the public were enrolled into separate groups which determined 59 criteria which covered the spectrum of factors related to both individual and population level disease burdens. The result highlights the difficulty in prioritizing zoonotic diseases because of the number of factors that need to be considered. Involving members of the public in the process drew attention to the narrowness and heterogeneity of expert which could limit the range of criteria considered during disease prioritization exercises.

Sometimes qualitative researchers conduct interviews with “naturally occurring” groups (for example, family members, or people who work together). Technically speaking, qualitative interviews with pre-existing groups are not focus groups (3). When interviewing members of pre-existing groups, important to be aware of how hierarchy within the group may affect the data. A farm hand, for example, could feel inhibited by the presence of a manager from the same property. Accordingly, qualitative researchers may conduct a series of one-to-one interviews rather than a group interview. Nonetheless, conducting one-to-one interviews in sequence cannot eliminate the potential for intimidation. Feelings of intimidation can diminish the quality of the interview data, but consenting to be interviewed could also pose risks to the participants. For instance, participants may worry because their herd or flock harbors a zoonotic infection, which could decrease the value and saleability of their livestock, if discovered. Hence qualitative researchers must proceed carefully when designing and conducting their studies, to minimize any potential for harm, and to balance potential benefits against any potential for harm.

**Storyboarding methodologies** encourages a different kind of research participation in that it enables lay-people to develop and communicate their knowledge about a specific issue using stories and non-textual media (80, 81). The approach centers on the creation and/or manipulation of visual elements (photographs, symbols, and drawings) or other materials such as plastic figurines, felt cloths, charts, and maps to develop an account of the social phenomena of interest to the researcher and participants. Alternatively, qualitative researchers may draw on techniques and processes from theater, such as role-plays (82, 83). The methodological focus is on stimulating a detailed representation of people's knowledge of how something happens, what things are valued and cared for within their communities, and their expectations as to the likely consequences of an event in a particular setting. The important feature is that the process works to centralize “story” as a key medium for sharing existing data and allow meaning-making to be directed by participants, increasing the likelihood that the results reflect their understandings (84).

To do this successfully, qualitative researchers need to be competent and confident in the use of the chosen non-textual media, be responsive to the needs of the research participants, and be prepared to be flexible in their approach (85). Hence qualitative researchers must allow those taking part to express their views, to the extent of molding the study according to their preferences and interests. However, a clear advantage of storyboard techniques is that they can break down traditional hierarchies (for example related to age differences or expertise of researcher and research subject); and they can facilitate communication by allowing the people to express their ideas or experiences in non-verbal ways, and on their own terms (86). Storyboard methods are particularly effective in bringing a focus on the social, temporal and spatial aspects of events or phenomena of interest—for example, the point of entry and likely transmission pathway of infectious disease outbreak in a remote or rural setting. By way of illustration, drawing on previous dog population surveys, preliminary qualitative interviews and a disease model (67, 87), storyboarding with communities in northern Australia allowed for the co-creation of knowledge about the potential impacts of a rabies outbreak, and to explore the feasibility and acceptability of different prevention and control strategies (88).

**Deliberative methodologies** involve members of the public or lay-people in a structured process to learn about, discuss and develop collective solutions to complex policy problems. Unlike approaches to social research that elicit participants' perspectives or experiences, deliberative methods revolve around a two-way exchange of information between members of the public, experts, and potentially, decision makers (89). Participants undergo a process of education about the problem under consideration, with an emphasis on promoting reason-based dialogue so they can expand their views through the consideration of factual information and the views of others. These features mean that deliberative methods can be used to provide public input to decision making around policy issues that cannot be resolved solely on the basis of technical information, but also require the consideration of public values. Deliberative engagement can also allow opportunities for members of the public to reframe public and health policy problems in terms that are important to them, and promote imaginative engagement with different policy options and potential futures (90). For example, one of the authors have run a series of Citizens'/Community juries convened in eastern Australia have involved citizens and members of affected communities in discussions about how best to manage the present and future risks of Hendra virus spill-over events in their local area (91). The outcomes indicate that members of the public are likely to strongly support ecological approaches to mitigating the risks of Hendra virus risks when informed of the relevant facts and dilemmas, but there is fundamental disagreement as to the most appropriate mechanisms to regulate land use change, and, thereby, create or better protect flying fox habitat.

Deliberative events construct a form of mini-public or interest group, such that composition of participants will determine what kind of claims of “representativeness” can be made about the verdict or outcome (89). A deliberative group comprised of people who are directly affected by the issue at hand (for example service users) will provide a different perspective (and a potentially different recommendation) to that of a group comprised of otherwise disinterested citizens who are not directly impacted by the matter under consideration. Clearly a small group of participants brought into deliberation cannot be politically or statistically representative of a much larger and diverse population. However, it is possible to aim for diversity in recruitment processes and to minimize selection bias. Participants can be selected based on socio-demographic criteria to ensure a diversity of perspectives is represented (92). Recruitment and selection of participants into deliberative groups should be organized based on the assumption that it is unrealistic to expect wide public understanding and deliberation, but it is possible to derive a sense of what informed and deliberative publics would advise from a smaller group (93).

Using deliberative methods is demanding—both in terms of time and resources. Finally, it is important to remember that different outcomes can and will occur when different groups of people are brought together to deliberate under highly similar conditions (89). Replication of an outcome across multiple events can add strength to the arguments and reasons put forward by participants at the end of each process. Divergence of outcomes between otherwise identical deliberative groups points to an enlarged range of positions and constituencies around the issue under consideration. Rather than trying to make all publics brought into deliberation respond in the same manner and come to the same conclusions, the goal of using these methods is to create the conditions where participants can engage in informed and reasoned discussions and make decisions and recommendation to policy-makers that authentically reflect their values and preferences (94).

## The Value of Qualitative Research Methods to Veterinary Epidemiology and Economics

Interactions between human and animal species are both beneficial and a source of risks to human health. The benefits and risks of our associations and interactions with other species are social patterned, such that small changes in how health and disease are distributed both within and across species boundaries. Public health has long been aware of the ways in which changes in how humans and animals co-exist can impact agricultural productivity and amplify burdens of disease (15). At the same time, our preoccupation with human health can render important dimensions of our relations with and reliance on animal species relatively invisible (95). The risks and benefits to human health of our interactions with and reliance on other species fold in on one another in complex ways such that just focusing on animal health can miss important dimensions of the bigger picture. For example, the mass culling of poultry conducted in response to highly pathogenic avian influenza outbreaks can result in stunting in children because of the loss of this vital source of protein (96, 97).

An overly medicalized model of the risks at the interface of human and animal health leaves insufficient space for a consideration of social well-being, and how this might be mediated in terms of relations with other species. The implications are that veterinary epidemiology and economics need to consider the relations between people (and how they engender or hinder health) and collaborate with and provide opportunities for other scientists with the necessary methodological expertise to both capture and take seriously how people think about their relationships with non-human species within the broader structures of social and economic systems that tie humans and animals together. Partnering with a social scientist can be a corrective to assuming that human-animal-environment interactions are somehow just natural systems, and understandable as if they exist independently of the social world that brings them into being (15, 98).

Against this background, qualitative research methods can be valuable to veterinary epidemiology and economics and instrumental to research processes and design in a number of ways. In the first instance qualitative research can describe, define and explain phenomena or areas that are not amenable to quantitative research methods. Spending time in the research setting, getting to know people and understand their points of view and daily experiences—which constitutes a form of ethnographic inquiry—is an important phase in the early stages of a research project. Familiarization and attention to what people do and what matters to them can put the core research question into context or generate novel questions for research that can be followed up by other quantitative and qualitative methodologies. Of course, until something is defined and classified appropriately it cannot be measured. Qualitative methods can be the foundation of efforts to enumerate variations in the relationship between features of the world—especially if the definition of what is being studied, a group of dogs at-large in public spaces (which might be a pack of strays, feral dogs, or free roaming dogs that are owned) for example, is unclear or ambiguous.

The second way qualitative methods can be valuable is as a supplementary or complementary study alongside quantitative work. Qualitative studies can be part of a validation process, in which a supplementary study is undertaken using a different method and the results are then compared for convergence. Or qualitative methods can be part of a multimethod approach which examines a particular phenomenon or topic on several different levels. The latter is not simply a matter of joining two techniques, and the former is not a case of tacking one on the end of a project. Though a survey may pick up the distribution of opinions of members of the public about an issue, a series of in-depth interviews will be required to access why people believe what they do, and how these beliefs inform their opinions. Different research settings and different methods allow access to different levels of knowledge and ways of acting in the social world. Combining methods can help to build a wider picture that can highlight hidden complexities and provide otherwise important context to the study findings.

### Potential Pitfalls and Misuse of Qualitative Research Methods

The ultimate goal of using qualitative methods is to produce a plausible and coherent explanation of the phenomenon under investigation. Poor quality analysis in qualitative health research is anecdotal and overly descriptive, and therefore lacks critical reflection or deep insight. All research depends on the application of some form of theory (5). Those using qualitative research methods need to be aware of the way in which different theoretical starting points can lead to different ways of doing research—which ultimately will determine the validity and usefulness of the research outcomes (12). Rigorous data collection technique and good quality analysis requires researchers who are appropriately trained, and most importantly, experienced in the methods and methodologies they are using. The reliability of study findings derived from most qualitative methods can be judged by the rigor and appropriateness of data collection and analyses processes. Demonstrating rigor and appropriateness requires the researcher to create and maintain meticulous and detailed records of interviews, observations, document searches, and the decisions (and their justification) made in each stage of the analysis. This record of the data and methods employed in collection and analysis should be able to stand independently so that another trained researcher could analyse the same data in the same way and come to the same basic conclusions.

The reliability of the analysis of qualitative data can be enhanced by organizing an independent assessment of transcripts by additional skilled qualitative researchers and comparing agreement between the analysts. Other validation strategies sometimes used in qualitative research are to present the study findings to the participants and see if they regard them as a reasonable account of their perspectives and experiences. Having more than one analyst can also provide assurances of consistency and that individual bias is not coloring data interpretation. It is important during the analyses to thoroughly explore negative or deviant cases, and to provide a coherent explanation of how the findings relate to, but are not invalidated by these variations. Social scientist also try to “triangulate” their findings by designing data collection processes in which evidence is deliberately sought from a wide range of different, independent sources (for example, comparing oral testimony with observations of peoples' behavior and textual sources such as reports from statutory bodies or news media). Because different groups are likely to have different perspectives, study findings also need to be interpreted in light of these other sources and forms of evidence.

Finally, the most significant pitfall for veterinary epidemiologist wanting to understand human motivations and actions using qualitative research methodologies is not having a trained social scientist on the investigator team. Qualitative methods are underpinned by both methodological and theoretical frameworks—and a thorough grounding in both is essential for study outcomes and findings to move beyond anecdote and basic description and achieve rigor in interpretation and explanation. Employing a method without properly considering or developing an understanding of the methodology means that the rationale for using a particular method is absent—as is the lens through which analysis takes place. Against this background it has been repeatedly observed that qualitative studies are largely absent from One Health research as currently construed, which has implications for the policy and real-world relevance of basic science and epidemiological studies that focus on the human-animal-environment interface (1, 2, 99, 100). By implication, we should prioritize efforts to recruit, train, and retain a cadre of qualitative researchers who specialize in One Health (98, 99). When performed well, qualitative methodologies can help veterinary economists and epidemiologists to develop impactful research questions, to create more accurate and contextually-relevant parameters for quantitative studies, and to develop policy recommendations and interventions that are attuned to the political and socio-cultural context of their implementation.

## Author Contributions

CD wrote the original draft. MR made substantial contributions to the writing of subsequent iterations. CD and MR contributed to subsequent redrafts. Both authors approved the final submission.

### Conflict of Interest

The authors declare that the research was conducted in the absence of any commercial or financial relationships that could be construed as a potential conflict of interest.
